# Insights on KP4 Killer Toxin-like Proteins of *Fusarium* Species in Interspecific Interactions

**DOI:** 10.3390/jof8090968

**Published:** 2022-09-16

**Authors:** Isabel Vicente, Giuseppe Quaratiello, Riccardo Baroncelli, Giovanni Vannacci, Sabrina Sarrocco

**Affiliations:** 1 Laboratory of Plant Pathology, Department of Agriculture, Food and Environment (DAFE), University of Pisa, Via del Borghetto 80, 56124 Pisa, Italy; 2 Laboratory of Plant Pathology, Department of Agricultural and Food Sciences (DISTAL), University of Bologna, Viale Fanin 46, 40127 Bologna, Italy

**Keywords:** mycotoxins, fungal competition, comparative genomics, gene expression, *Fusarium graminearum*, *Trichoderma gamsii*

## Abstract

KP4 killer toxins are secreted proteins that inhibit cell growth and induce cell death in target organisms. In *Fusarium graminearum*, KP4-like (KP4L) proteins contribute to fungal virulence in wheat seedling rot and are expressed during Fusarium head blight development. However, fungal KP4L proteins are also hypothesized to support fungal antagonism by permeabilizing cell walls of competing fungi to enable penetration of toxic compounds. Here, we report the differential expression patterns of *F. graminearum* KP4L genes (*Fgkp4l-1*, *-2*, *-3* and *-4*) in a competitive interaction, using *Trichoderma gamsii* as the antagonist. The results from dual cultures indicate that *Fgkp4l-3* and *Fgkp4l-4* could participate in the recognition at the distance of the antagonist, while all *Fgkp4l* genes were highly activated in the pathogen during the physical interaction of both fungi. Only *Fgkp4l-4* was up-regulated during the interaction with *T. gamsii* in wheat spikes. This suggests the KP4L proteins could participate in supporting *F. graminearum* interspecific interactions, even in living plant tissues. The distribution of KP4L orthologous within the genus *Fusarium* revealed they are more represented in species with broad host-plant range than in host-specific species. Phylogeny inferred provides evidence that KP4L genes evolved through gene duplications, gene loss and sequence diversification in the genus *Fusarium*.

## 1. Introduction

The genus *Fusarium* is one of the most important groups of phytopathogenic fungi affecting a wide range of crops in all climatic zones around the globe [1]. Some *Fusarium* species are responsible for agriculturally important plant diseases, such as Fusarium head blight (FHB) in cereals, and a variety of wilt and stem root diseases causing enormous economic losses [2]. FHB is caused by phylogenetically diverse *Fusarium* species that are grouped into the FHB Species Complex—FHBSC [3], being *F. graminearum* and *F. culmorum* considered among the most prevalent and aggressive [4]. FHB causes significant yield losses with a concomitant reduction of grain quality due to the accumulation of mycotoxins, fungal secondary metabolites very harmful to humans and their livestock [5]. The use of beneficial fungi represents an interesting strategy to reduce the impact of FHB in favor of more sustainable agricultural practices [6,7]. During the last ten years, *Trichoderma gamsii* T6085 has been used both in lab and field trials for the biocontrol of FHB, acting as an antagonist, a mycoparasite and a competitor for natural substrates, being able to reduce *F. graminearum* and *F. culmorum* growth [8,9,10]. A recent transcriptomic survey addressing the molecular dialogue between *T. gamsii* T6085 and *F. graminearum* ITEM 124 revealed fine-regulated crosstalk during the non-self-recognition of the two fungi genes encoding proteins with homology to the KP4 killer toxins were highly up-regulated in the pathogen [11].

Killer toxins are secreted proteins able to inhibit cell growth and to trigger cell death by blocking the voltage-gated calcium channels and other elements of the calcium-dependent signaling pathways in target organisms [12,13,14]. First described in the basidiomycete *Ustilago maydis* [15], KP4 proteins have been also found in the yeasts *Saccharomyces cerevisiae* [16] and *Kluyveromyces* spp. [17], in the moss *Physcomitrella patens* [18], as well as in many Ascomycota species [18,19,20].

In *Fusarium*, homologues of KP4 genes (KP4-like—KP4L) have been identified in isolates belonging to 12 species, ranging from 4 to 6 genes per genome [11,18,20,21]. The genome of *F. verticillioides* 7600, contains six KP4L genes showing the typical KP4 conserved domain (Pfam09044) [18]. Three of these genes (i.e., *kp4l-1*, *kp4l-2*, *kp4l-3*) were found included in a 3.4 Kb cluster, while the others (i.e., *kp4l-4*, *kp4l-5*, *kp4l-6*) are located elsewhere in the genome [18].

In *F. graminearum* PH-1, three small, secreted and cysteine-rich proteins belonging to the KP4 superfamily (i.e., KP4L-1, KP4L-2 and KP4L-3) were found expressed during fungal-wheat spikes interaction [21], and later, the heterodimeric protein KP4L-4 was identified by a genomic database search [20]. Phylogenetic analysis of KP4L proteins of 85 fungal species, including *Fusarium* spp. and other organisms, suggesting a cross-kingdom horizontal gene transfer as the mechanism responsible for KP4 gene diversification in eukaryotes [20].

The involvement of KP4L genes in fungal virulence has been highlighted, playing an important role during the host-plant infection process. The expression of the *kp4l-2* gene of *F. verticillioides* was found up-regulated in the presence of maize endosperm and embryo, thus suggesting a fungal virulence factor [18]. Similarly, the expression of all the four KP4L-encoding genes of *F. graminearum* PH-1 was induced during the infection of wheat seedlings, while only *kp4l-1*, *kp4l-2* and *kp4l-3* were expressed during FHB development (4 h to 14 dpi) both in susceptible and resistant wheat cultivars [20]. Nevertheless, KP4 proteins are also hypothesized to support fungal antagonism by permeabilizing the cell walls of competing fungi to enable penetration of toxic compounds [22]. Induction of KP4L-encoding genes during interspecific fungal interactions and functional studies in filamentous fungi support this idea and also suggest their expression is responsive to stress conditions [20,23].

In the present work, we report the identification and the phylogenetic relationships of KP4L genes of 29 isolates belonging to 15 *Fusarium* species, providing evidence that KP4L genes evolved through gene duplications, gene loss and sequence diversification in *Fusarium*. We also present differential expression patterns of four KP4L genes of *F. graminearum* (*Fgkp4l*) in a competitive fungus-fungus interaction using *T. gamsii* as the antagonist. Results from time-course experiments performed in dual cultures and wheat spikes revealed that *Fgkp4l* genes are differentially expressed during the recognition at the distance of the antagonist and are highly up-regulated during the physical interaction of both fungi, thus suggesting a possible role of KP4L proteins in supporting *F. graminearum* interspecific interactions.

## 2. Materials and Methods

### 2.1. Fungal and Plant Material

*Trichoderma gamsii* T6085 was isolated in Crimea (Ukraine) from uncultivated soil [8]. *Fusarium graminearum* ITEM 124 (*Fg*), isolated from rice, belonged to the fungal collection of the CNR-ISPA (Bari, Italy) and was kindly given by Antonio Moretti [24]. Fungi were maintained at 4 °C under mineral oil on Potato Dextrose Agar (PDA) (Sigma-Aldrich, Milan, Italy) for long-term storage and were grown on PDA at 24 °C, 12 h light/12 h darkness when actively growing colonies were needed. The pathogen was regularly passed through the host plant to maintain its virulence.

Seeds from *Triticum aestivum* cv. USU-Apogee (soft wheat) were surface-sterilized with NaClO (0.6% active chlorine) for 3 min under gently shaking, then washed three times for 10 min each with sterile distilled water and stored at 4 °C for three days for vernalization. Seeds were then sown in pots in a commercial potting mix and incubated in a growth chamber (Photoperiod of 16 h light at 22 °C/8 h darkness at 20 °C).

### 2.2. Dual Culture Tests of F. graminearum with T. gamsii

Dual culture tests of *F. graminearum* and *T. gamsii* were performed in Petri dishes (90 mm diameter) containing PDA (Sigma-Aldrich, Milan, Italy) overlaid with a sterile cellophane membrane (PT-35GR, 90 mm). The plates were inoculated with two rectangular strips (length 50 mm, width 5 mm) of PDA 2X colonized by actively growing mycelium of the two isolates. The strips were placed 7 cm apart. The experiment consisted of two theses: *F. graminearum* vs. *T. gamsii* (*F. graminearum* non-self-interaction) and *F. graminearum* vs. itself (*F. graminearum* self-interaction), with the last used as a control. Plates were incubated at 24 °C in a 12 h light/12 h darkness cycle. The mycelium of *F. graminearum* were collected at three different phases of the interaction (Figure S1): early-sensing phase, when the two colonies were 15 mm apart; sensing phase, when the two colonies were at 5–8 mm apart; and after-contact phase, 24 h after the contact between the two colonies, when they started to overgrow. Three biological replicates were included for each thesis and each sampling point, each replicate consisting of six plates. Each sample from the early and sensing phases, in both self- and non-self-conditions, consisted of a 5 mm strip of mycelium of *F. graminearum* collected from the edge of the colony facing the opposite colony. In contrast, each sample from the after-contact phase consisted of a 10 mm strip of mycelium collected from the contact zone between colonies. Mycelial strips collected from each of the six plates of the same replicate were merged together, frozen in liquid N_2_ and stored at −80 °C until RNA extraction.

### 2.3. Interaction of F. graminearum with T. gamsii on Wheat Spikes

Sterilized and vernalized wheat seeds were grown until plants reached the anthesis stage (five weeks). The experiment consisted of two theses: wheat spikes inoculated with *F. graminearum* + *T. gamsii* or with *F. graminearum* alone. For *T. gamsii* inoculation, conidia were collected from one-week-old PDA plates with 20 mL of sterile 0.01% Tween-80 solution (Carlo Erba, Milan, Italy), and a 10^7^ mL^−1^ spore suspension was sprayed on the wheat spikes of the *F. graminearum* + *T. gamsii* thesis. Plants were covered with a white bag, previously moistened inside with water to maintain humidity, and incubated in a growth chamber for 48 h. After 48 h, the white bag was removed for plant aeration and *F. graminearum* inoculation. For inoculation of the pathogen, conidia of *F. graminearum* were collected from 2-week-old PDA plates with 20 mL of sterile 0.01% Tween-80 solution, and a 10^5^ mL^−1^ spore suspension was sprayed on wheat spikes of both *F. graminearum* alone and *F. graminearum* + *T. gamsii* theses. The plants were then covered again with a white bag, and a black bag was also placed to facilitate the infection by the pathogen. Plants were incubated in a growth chamber in the same conditions as described above. After 24 h, the black bags were removed, the plants were put back into the chamber, and, after 24 h, the white bags were completely removed [25]. Three biological replicates, each including four plants, were inoculated per each thesis. Spikes colonized by the fungi were collected at 7 and 14 days after inoculation (dpi) with the pathogen, frozen in liquid N_2_ and stored at −80 °C until RNA extraction.

### 2.4. Analysis of Expression of Fgkp4l Genes

#### 2.4.1. RNA Extraction and cDNA Synthesis

The mycelium of *F. graminearum* collected from the dual cultures was ground in liquid N_2_ using a pre-chilled mortar and pestle. Samples containing 100 mg of powder were used for total RNA extraction with the RNeasy^®^ Plant Mini Kit (Qiagen, Milan, Italy), according to the manufacturer’s instructions. The wheat spikes colonized by the fungi were ground in liquid N_2_ using pre-chilled mortar and pestle, and samples containing 300 mg of powder were used for total RNA extraction according to the method described by Logemann et al. [26]. RNA integrity was checked by agarose electrophoresis in TBE 0.5X. RNA samples were incubated with DNase I (DNase I Amplification Grade, AMPD1 Sigma-Aldrich, Milan, Italy) for gDNA removal, according to the manufacturer’s instructions. A total of 400 ng of RNA were used for cDNA synthesis using Maxima First Strand cDNA synthesis kit (K1642, Thermo Scientific, Milan, Italy) according to the manufacturer’s instructions.

#### 2.4.2. Gene Expression Analyses

The relative gene expression of *Fgkp4l-1*, *Fgkp4l-2*, *Fgkp4l-3* and *Fgkp4l-4* was analyzed by quantitative Real-Time PCR (qRT-PCR) using Rotor-Gene Q cycler (Qiagen, Milan, Italy). Reactions were set up with 10 ng of cDNA, 10 µL of QuantiNova SYBER^®^ Green PCR Master Mix 2X (Qiagen, Milan, Italy), 1.4 µL of each primer (0.7 µM) and Nuclease-Free water up to 20 µL of the final volume. DNase I-treated RNA samples and Nuclease-Free water were used as non-template-control reactions. PCR reactions were performed in triplicate per each biological replicate under the following conditions: initial activation, 95 °C, 2 min; 40 cycles of denaturation, 95 °C for 5 s and combined annealing/extension, 60 °C for 10 s. Threshold cycles (Cts) were calculated with Rotor-Gene Q Series Software v2.3.1 (Qiagen, Milan, Italy) using the actin gene as endogenous control, which was selected among other housekeeping genes (β-tubulin and transcription elongation factor-1 genes) due to its expression stability in all the samples according to the MIQE guidelines [27]. Data were expressed as 2^−ΔΔCt^ [28]. The values from three biological replicates were consistent and used for ANOVA and Tukey’s post-hoc test statistical analyses, using SYSTAT© v.13.2 software (Systat Software, San José, CA, USA) (*p* ≤ 0.05). The primers used for gene expression analysis (Table S1) were checked for efficiency, dimer formation and possible cross-amplification of either wheat or *T. gamsii* transcripts.

### 2.5. Identification and Phylogenetics of KP4L Proteins of Fusarium *spp.*

The genomes with gene prediction and annotation, available in public databases (National Center for Biotechnology Information—NCBI—[29]) of 29 isolates belonging to 15 *Fusarium* species and of *T. gamsii* T6085 were used for computational analyses (Table S2 [30,31,32,33,34,35,36,37,38,39,40,41,42,43,44,45,46,47,48,49]). To determine the phylogenetic relations of *Fusarium* spp., full proteomes were analyzed with OrthoFinder v2.5.4 (University of Oxford, Oxford, UK) [50]. Single copy genes present in all the genomes were extracted, aligned with MAFFT v7.450 (University of Osaka, Suita, Japan) [51] and concatenated using Geneious Prime^®^ v2022.1.1 (Dotmatics, Bishop’s Storltford, UK) [52]. Multi-locus alignment was used as input for phylogenetic analysis, using FasTree v2.1.11 (University of California, Berkeley, CA, USA) [53].

The KP4L proteins were identified in *Fusarium* proteomes by using the 127-amino acid sequence of the *U. maydis* KP4 protein (UmKP4) (GenBank accession number Q90121) [54] as a query for BLASTp searches against the proteomes of selected species with a cut-off value of 10^−5^ (E-5). KP4L proteins were also retrieved using InterProScan [55] by searching the KP4 conserved domain entries IPR015131 and Pfam09044 in *Fusarium* proteomes. KP4L proteins of *Fusarium* spp. were further classified into different groups, according to the sequence similarity shared with known KP4L proteins of *F. graminearum* [11,20] and their pairwise intersection distances using Intervene (University of Oslo, Oslo, Norway) [56]. The KP4 conserved domains were extracted from KP4L proteins using the Geneious Prime^®^ v2022.1.1 software (Dotmatics, Bishop’s Storltford, UK) [52]. The KP4 domains of each protein group were aligned separately by MAFFT v7.450 (University of Osaka, Suita, Japan) [51], considering species within and outside the FHBSC as a single group or as independent groups. The resulting consensus sequences were further aligned with the UmKP4. The two non-identical KP4 domains (D1 and D2) of KP4L-4 proteins were independently considered for the alignments. For phylogenetic analysis, KP4 domains were used for multiple-alignment using MAFFT v7.450 (University of Osaka, Suita, Japan) [51] along with the UmKP4, used here as an outgroup. Multiple alignments were used to construct a phylogenetic tree using MEGA-X. The best substitution model was obtained using ProtTest (University of Vigo, Vigo, Spain) [57]. The phylogenetic tree was reconstructed using the WAG + I evolutionary model [58]. The posterior probabilities and bootstrap values threshold were 50%.

## 3. Results

### 3.1. Differential Expression of Fgkp4l Genes in Dual Culture Tests with T. gamsii

**Figure 1:**
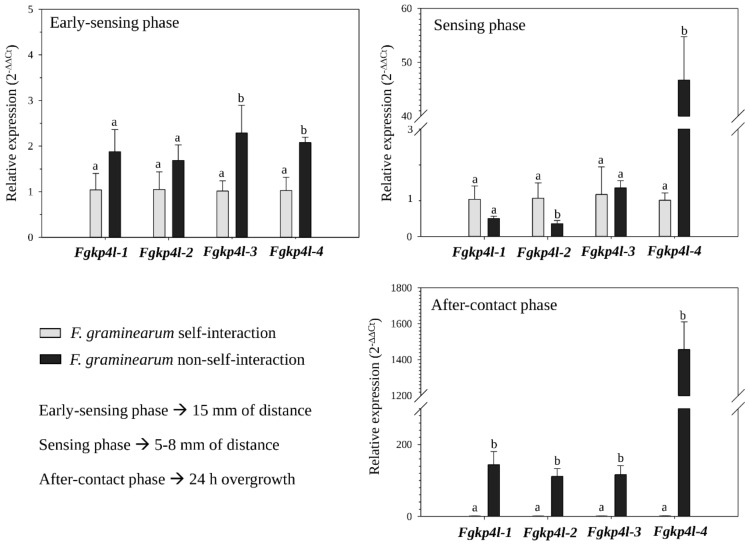
Gene expression profiles of *Fgkp4l* genes in dual cultures with *T. gamsii*. Total RNA was extracted from mycelium of *F. graminearum* grown on PDA vs. itself (self-interaction, basal condition 2^−ΔΔCt^ = 1) and vs. *T. gamsii* (non-self-interaction) at three different sampling times of the interaction, corresponding to the early-sensing phase (15 mm of distance between fungal colonies), the sensing phase (5–8 mm of distance between fungal colonies) and the after-contact phase (24 h of overgrowth). The actin gene was used as an endogenous control for data normalization. The values are the means of the three independent biological replicates with the corresponding standard deviation. Fold change in sample relative to control is expressed as 2^−ΔΔCt^. The different letters correspond to statistically different values for *p* ≤ 0.05 (ANOVA).

The qRT-PCR results indicated that the four *Fgkp4l* genes were expressed similarly (22 to 25 Cts) in *F. graminearum* grown vs. itself on PDA (self-interaction), regardless of the sampling time. When *F. graminearum* was grown on PDA with *T. gamsii* (non-self-interaction), the expression levels of *Fgkp4l* genes varied depending on the distance between the two fungal colonies (Figure 1). Although the expression of some *Fgkp4l* genes was found differentially regulated in the early and sensing phases of the interaction at a distance between both fungi (*Fgkp4l-3* = 2.29-fold up-regulated in the early phase; *Fgkp4l-4* = 2.08-fold and 46.70-fold up-regulated during the early and the sensing phases, respectively; *Fgkp4l-2* = 0.36-fold down-regulated at the sensing phase), it was during the physical contact between the colonies when all the four *Fgkp4l* genes were strongly activated in the pathogen (143.89-, 111.45-, 116.30-, 1456.80-fold up-regulation for *Fgkp4l-1*, *-2*, *-3* and *-4*, respectively). These results suggest that the *Fgkp4l* genes may have a role in *F. graminearum*-fungus interactions, especially when the pathogen is in close contact with its antagonist. Among all the genes, *Fgkp4l-4* seems to have an important role even in the non-self-recognition at distance, since its expression increases very rapidly from the early phase of the interaction with *T. gamsii* reaching higher transcriptional values as the distance between both fungi decreased.

**Figure 2:**
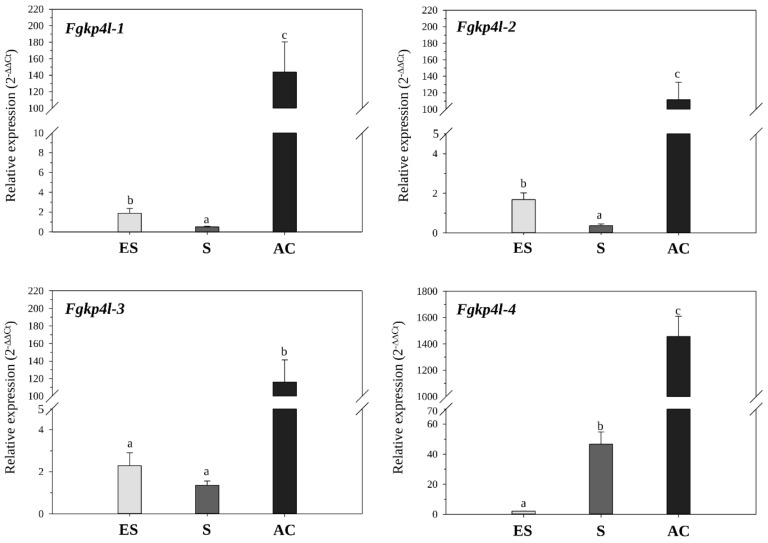
Expression profiles of each *Fgkp4l* gene during the three phases of interaction with *T. **gamsii*****.** The bars represent means of three biological replicates with the corresponding standard deviation of values from the non-self-interaction condition obtained during three different sampling points: early-sensing phase (ES), 15 mm of distance between fungal colonies; sensing phase (S), 5–8 mm of the distance between colonies; after-contact phase (AC), 24 h overgrowth. The different letters correspond to statistically different values for *p* ≤ 0.05 (ANOVA and Tukey’s test).

Comparison of the fold-change values of each *Fgkp4l* gene in the non-self-interaction among the three phases revealed three different expression profiles (Figure 2). While the expression of *Fgkp4l-4* increased sharply from the early phase up until after contact, the expression of *Fgkp4l-3* did not change during the interaction at a distance, but that of *Fgkp4l-1* and *-2* slightly decreased between the early and the sensing phases. This indicates that the *Fgkp4l* genes respond differently to the signals released by the facing colony during their interactions at a distance.

### 3.2. Differential Expression of Fgkp4l Genes during the Interaction with T. gamsii on Wheat Spikes

**Figure 3:**
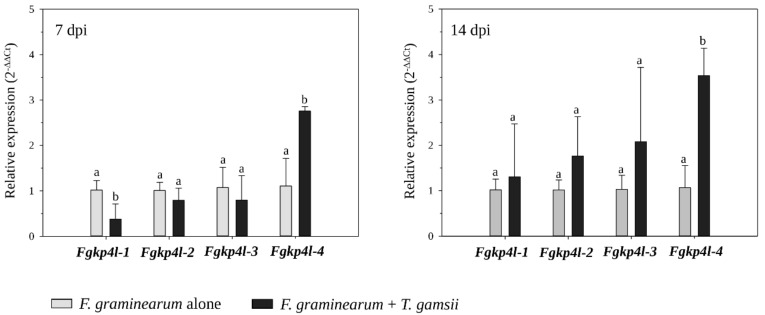
Gene expression profiles of *Fgkp4l* genes during the interaction with *T. gamsii* on wheat spikes. Total RNA was extracted from wheat spikes colonized by *F. gramineraum* alone (basal condition 2^−ΔΔCt^ = 1) and colonized by both the pathogen and *T. gamsii* at 7 dpi and 14 dpi with the pathogen. The actin gene was used as an endogenous control for data normalization. The values are the mean of three independent biological replicates with the corresponding standard deviation. Fold change in sample relative to control is expressed as 2^−ΔΔCt^. The different letters correspond to statistically different values for *p* ≤ 0.05 (ANOVA).

The relative expression of *Fgkp4l* genes was evaluated using material collected from wheat spikes inoculated with both *F. graminearum* and *T. gamsii*—at 7 and 14 dpi with the pathogen, using wheat spikes inoculated with the pathogen alone in the same conditions as the controls (Figure 3).

Results showed that only *Fgkp4l-4* gene expression was up-regulated both at 7 and 14 dpi (2.76- and 3.53-fold, respectively), while *Fgkp4l-1* expression was 0.38-fold down-regulated when interacting with *T. gamsii* in wheat spikes at 7 dpi. This could support the role of *Fgkp4l-4* gene in *F. graminearum*-fungus interactions, even on living plant parts. A comparison of the fold-change values of each gene during the tri-trophic interaction between 7 and 14 dpi revealed no significant changes in gene expression over time.

### 3.3. Identification and Distribution of KP4L Proteins in Fusarium Genomes

**Figure 4:**
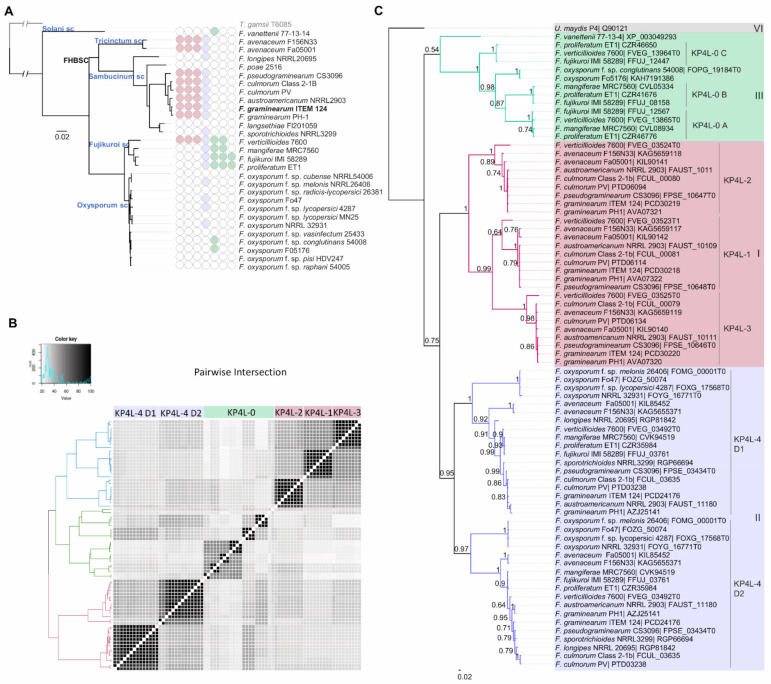
Distribution and phylogenetic relationships of KP4L proteins in the genus *Fusarium***.** (**A**) Phylogenetic relationships of *Fusarium* spp. species were included into different species complexes according to the taxonomy described by O’Donnell et al. [38] and Summerell [1]. All nodes were supported by bootstrap values of 100%. The KP4L-gene content per each species is shown in colored dots on the branches of the tree of each species: Group I (pink color) including KP4L-1, -2 and -3, Group II (blue color) including KP4L4, Group III (green color) including KP4L-0 A, B and C, and Group VI (grey color) including the *U. maydis* KP4. (**B**) A pairwise intersection heat map of KP4L proteins was calculated using Intervene (University of Oslo, Oslo, Norway) [56]. (**C**) Phylogenetic characterization of *Fusarium* spp. KP4L proteins. Proteins were classified according to the groups as described by Lu and Faris [20]: Group I (pink color), Group II (blue color), Group III (green color) and Group VI (grey color).

*Fusarium* spp. here considered fell into five different phylogenetic species complexes: *Tricinctum* and *Sambucinum* complexes (corresponding to the FHBSC), and the *Solani*, *Fujikuroi* and *Oxysporum* species complex (Figure 4A). A total of 58 KP4L proteins with similarity with the UmKP4 protein were inferred from the genomes of 21 isolates belonging to 13 *Fusarium* species. Each protein consisted of 125 to 283 residues and contained a 16–21 residues N-terminal signal peptide sequence. The InterProScan analysis confirmed that all the proteins contained the KP4 conserved domain (IPR015131 and Pfam09044) with 18 proteins containing a heterodimeric KP4 domain. Based on the amino acidic sequence similarity shared with known KP4L proteins of *F. graminearum* [11,20] and at their pairwise intersection distances, the proteins were classified into three different groups: one group containing KP4L-1 (9 proteins), KP4L-2 (9 proteins) and KP4L-3 (9 proteins), one group containing KP4L-4 (18 proteins) and a group named KP4L-0 containing 13 proteins with low sequence similarity (36–47%) with known KP4L proteins (Figure 4B).

The KP4L gene-family size ranged from one to four genes per each *Fusarium* genome (Figure 4A), with the exception of *F. verticillioides*, which contains two additional genes (KP4L-5 and KP4L-6, here classified as KP4L-0), as previously reported [18]. KP4L-1, KP4L-2 and KP4L-3 were only present in species of the FHBSC and in *F. verticillioides*, while KP4L-0 were only found in species outside the FHBSC. In particular, KP4L-0 were found highly represented in the species belonging to the *Fujikuroi* species complex, which harbor between two to three gene copies per genome. Instead, KP4L-4 is present in all the phylogenetic groups, with the exception of *F. vanettenii* from the *Solani* species complex. No KP4L proteins were found in *F. langsethiae* and *F. poae* from the FHBSC, and in six *F. oxysporum* genomes. While KP4L-1, -2 and -3 are mostly represented in species causing FHB and KP4L-0 in species infecting mainly maize, KP4L proteins are almost absent in species of the *Oxysporum* species complex.

Intraspecific variability of the KP4L proteins was evaluated in *F. graminearum*, *F. culmorum*, *F. avenaceum* and *F. oxysporum* (Table S3a). In *F. culmorum* and *F. graminearum* KP4L proteins were 100% identical among isolates, except for FgKP4L-4, which were 95% similar in *F. graminearum*. *F. avenaceum* KP4L proteins showed a remarkable diversity among isolates (96 to 99% similarity). In *F. oxysporum*, only the KP4L-4 proteins were found to have a 99% and 100% sequence similarity within the D1 and D2 domains, respectively. In terms of interspecific variability (Table S3b), KP4L proteins were more conserved within FHBSC (79–99% similarity) than outside the complex (56–73%), with an overall sequence similarity of 56–94% among all *Fusarium* spp. Comparison between all *Fusarium* KP4L proteins (i.e., KP4L-0, -1, -2, -3 and -4) and the UmKP4 showed they share a 44% similarity. In particular, a comparison of single KP4L groups (i.e., KP4L-0, -1, -2, -3 or -4) with the UmKP4 showed that proteins of FHBSC species were more conserved with UmKP4 (36–41% similarity) than proteins of species outside the complex (30–39% similarity) (Table S3c), being KP4L-4 proteins the less conserved in both cases.

### 3.4. Phylogenetics of KP4L Proteins in Fusarium *spp.*

Phylogenetic analysis revealed a remarkable polymorphism within the KP4L protein family in *Fusarium* (Figure 4C). KP4L proteins are divided into three different major groups corresponding to those described by Lu and Faris [20]: Group I containing KP4L-1, KP4L-2 and KP4L-3; Group II containing D1 and D2 domains of KP4L-4; and Group III containing KP4L-0. Sequence comparison of proteins from Group III with those belonging to Groups I and II showed they share low sequence similarity (37–47%). Results suggest that the duplication of the ancestral gene originated two copies from which all the KP4L proteins of *Fusarium* spp. would have derived. One of these copies underwent further gene duplications, leading to genes encoding KP4L-1, KP4L-2, KP4L-3 and KP4L-4 proteins (Groups I and II). Within Group II, heterodimers D1 and D2 clustered separately, suggesting an internal domain duplication of the KP4L-4 ancestor gene. Instead, the second copy originated genes encoding KP4L-0 proteins (Group III). Some species contain a single KP4L-0 protein (*F. vanetteni*, *F. oxysporum* f. sp. *conglutinans* and *F. oxysporum* Fo5176), while other species contain either two (*F. mangiferae* and *F. verticillioides*) or three (*F. proliferatum* and *F. fujikuroi*) KP4L-0 proteins. These three scenarios suggest at least two independent gene duplications events from the ancestral KP4L-0-encoding gene that originated KP4L-0 A, KP4L-0 B and KP4L-0 C paralogs in the clade Asian of the *Fujikuroi* Species Complex. The presence of KP4L-0 A, B and C in *F. proliferatum* and *F. fujikuroi* and the absence of C and B in *F. mangiferae* and in *F. verticillioides*, respectively, suggest loss of C and B in these last species. Similarly, the presence of KP4L-1, -2 and -3 in all the species belonging to the *Sambucinum* species complex, with the exception of *F. longipes* and *F. poae* suggests a loss of these genes in both species. No isolated KP4L-1, -2 or -3 were found in *Fusarium* genomes, suggesting co-inheritance of these three genes.

## 4. Discussion

The KP4 protein is one of the three virally encoded killer-toxin proteins firstly identified in certain isolates of the corn smut fungus *U. maydis* [20], and homologues of the UmKP4 (KP4L) have been previously inferred in isolates belonging to 12 *Fusarium* species [11,18,20,21].

In our study, a total of 58 inferred proteins were found distributed across 21 out of 29 *Fusarium* genomes, indicating that KP4L proteins are widely spread across the genus. However, KP4L are more represented in *Fusarium* species sharing a similar and broad host-plant range than in host-specific species, such as those of the *Oxysporum* species complex. Fungal pathogens specialized in infecting specific plant host could be under a lesser competitive pressure compared to host-generalist species than can coexist and compete each other for infection sites, as observed in species of the FHBSC [59]. Within strong competitive environments, climatic conditions and/or pathogenic alliances can confer advantages to certain species, while exploitation of specific genetic traits would be determinant to overcoming competing species. For example, in yeast, killer toxins are secreted as a mechanism of interference competition against yeasts inhabiting the same niche [60]. In this context, the KP4L distribution in *Fusarium* could be related to differences in the competitive pressure existing above the species. Thus, the KPL4 proteins would provide *Fusarium* species more opportunities to prevail in strong competitive niches. Also, the intraspecific and isolate-specific variability found in the KP4L gene content in *F. oxysporum* evidences the effect of niche differentiation in KP4L distribution within *Fusarium*.

A comparison of *Fusarium* KP4L proteins with the UmKP4 indicates that KP4 proteins are actually quite conserved between Ascomycota and Basidiomycota, thus suggesting similar biological functions. Our phylogenetic analysis revealed a remarkable polymorphism within the KP4L protein family in *Fusarium*. Experimental evidence has indicated that the KP4 progenitor originated in the fungal kingdom, and it has been speculated that ancestral *Fusarium* species acquired the progenitor genes from an ancestral *Aspergillus* species [18].

According to our results, a duplication of the ancestral gene originated two copies from which all the KP4L proteins of *Fusarium* would have arisen. One of these copies diversified into KP4L-1, KP4L-2, KP4L-3 and KP4L-4 through gene duplications, while the second copy led to genes encoding KP4L-0 proteins that may constitute a new class of KP4L proteins with different biological functions. Considering that KP4L-0 are only present in species outside the FHBSC, while KP4L-1, -2, -3 are mainly represented in species of the FHBSC, protein sequence diversification could be related to ecologic divergences existing between both groups of species. The UmKP4 showed higher sequence similarity with FHBSC KP4L proteins than with proteins of species outside the complex, which may support the functional differentiation of KP4L proteins and the evolution of different biological functions. It has been proposed that activation of *kp4l-1*, *-2* and *-3* genes during FHB development could be associated with host-derived stress signals since deletion of the entire genomic region comprising these genes in *F. graminearum* did not reduce fungal virulence in FHB [20]. It could be of interest to further investigate the role/s of KP4L-1, -2 and -3 in the ecology of FHB causal agents when interacting with each other.

As reported in other fungi, divergence observed for heterodimers D1 and D2 of *Fusarium* KP4L-4 proteins suggests an internal domain duplication of the KP4L-4 ancestor gene and posterior parallel evolution of both domains that have been maintained during the speciation process [18,20]. On the basis of these findings, the diversity observed within the KP4L protein family of *Fusarium* spp. is likely the result of gene duplications, gene loss and sequence diversification of the ancestral gene, which is in agreement with the evolutionary model proposed for killer toxin-like-encoding genes in other Ascomycota species [61].

Although the KP4L proteins act as virulence factors during host-plant infection processes [18,20,62] they are also hypothesized to play a pivotal role during fungus-fungus interactions by facilitating penetration of antifungal compounds into the cells of competing fungi [22]. Mycotoxigenic *Fusaria* secrete toxic compounds as a mechanism to compete with other organisms [63], as demonstrated by a transcriptomic analysis applied to the *Fusarium odoratissimum—Trichoderma guizohuense* interaction [64]. The expansion of KP4L genes observed in *Fusarium* and *Trichoderma* genomes suggests they may be relevant in the ecology of both genera [18]. Zapparata et al. reported the induction of KP4 genes of *F. graminearum* (*Fgkp4l*) during the non-self-recognition at distance using *T. gamsii* as the antagonist, suggesting these genes actively support competitive interactions established by the pathogen with other fungi [11].

In our study, the experimental time frame of Zapparata et al. [11] was expanded to both the early and late phases of the *F. graminearum*-*T. gamsii* interaction. Results indicate that the four *Fgkp4l* genes may have a role in the competitive interaction *F. graminearum* establishes with *T. gamsii* when the pathogen is physically interacting with its antagonist, while *Fgkp4l-3* and *Fgkp4l-4* could also be involved in the first stages of non-self-recognition at a distance. The results also indicate that the physical contact between the fungi is required for a strong *Fgkp4l* gene activation and gene co-expression. This suggests the DAMP (Damage-Associated Molecular Pattern) molecules and/or the sensing of cell wall components are likely involved in *Fgkp4l* gene induction, as observed for other KP4 chitinases [65].

The induction of KP4 proteins during other fungal self-interactions indicates they probably support nutrient acquisition or cell wall-remodeling processes [23], which could explain the basal expression of *Fgkp4l* genes found here in the control plates. *Fgkp4l-4* showed the highest transcriptional levels compared to *-1*, *-2* and *-3* in all the sampling times, and was also up-regulated in spikes with *T. gamsii*, which suggests an important role of this gene in *F. graminearum* interspecific interactions even if on living plant tissues. The gradual increase observed in *Fgkp4l-4* expression as the distance between colonies decreased indicates this gene may respond to signals released by the facing colony in a gradient-dependent way. The lack of *Fgkp4l* gene co-expression during the sensing phase of the interaction is in contrast with what was observed by Zapparata et al. [11], who found all the *Fgkp4l* genes up-regulated in similar experimental conditions. The range of the sensing phase varied from 5 to 8 mm of the distance between the colonies; thus, different results could be explained by slight differences in the sampling time between both experiments.

The co-regulation of *Fgkp4l* genes observed when both fungi were in close contact suggests they may be controlled by certain common regulatory pathways. However, the different expression profiles observed during the interaction at a distance on PDA and in wheat spikes with *T. gamsii* suggest a more complex regulatory network of these genes in *Fusarium*-fungus interplays. In the conditions tested here, *Fgkp4l* genes respond differently to the same signals, and/or are regulated by different signals during the fungal interaction at distance. These signals could be either volatile or soluble compounds released in the substrate by the facing colony, even chemical changes induced in the substrate as a result of the fungal growth. Non-common expression patterns of *Fgkp4l* genes have also been observed in mycelia grown in axenic cultures, in response to certain stress conditions and during fungal plant infection [20]. These authors raised the hypothesis that different expression patterns could be due to the variations found in the 5′ untranslated region of individual *Fgkp4l* mRNA sequences. Nevertheless, it could also be that the expression patterns of the KP4L proteins in fungus–fungus interactions are highly dependent on the interacting species [23] and on the stress status of both partners [20].

The *Fgkp4l-1*, *-2* and *-3* genes are located contiguous in the genomes of *F. graminearum* ITEM 124 [11] and PH-1 [20], which, together with the distribution of these genes in *Fusarium* genomes here observed, supports they originated from a common ancestor and are inherited together. The deletion of the whole 5Kb cluster in *F. graminearum* PH-1 resulted in reduced fungal virulence in wheat seedling rot [20]. Furthermore, the recombinant KP4L-2 protein of *F. graminearum* PH-1 was able to inhibit the growth of wheat root/shoot seedlings in in vitro experiments [20]. The co-regulation of *Fgkp4l-1*, *Fgkp4l-2* and *Fgkp4l-3*, together with the evidence that their encoded proteins were found to be 100% identical between the isolates PH-1 and ITEM 124, indicate they probably have the same role in fungal virulence towards the host plant.

On the basis of these results, *F. graminearum* could secrete KP4L proteins as a strategy to counteract *T. gamsii* in response to stress signals and/or signals secreted by the antagonist in a gradient-dependent way and, probably, in response to other molecules released during the mycoparasitic interaction between both fungi. This is in line with what was hypothesized by Tzelepis and Karlsson [23], who discussed a possible role of fungal killer toxin-like proteins in the self-protection against the action of presumably dangerous enzymes released by competitors.

Here, we provided some evidence that *Fgkp4l* genes could be involved in *F. graminearum*-fungus interactions. Further studies involving the use of KP4L deletion mutants are needed to determine the role of these genes in the physiology of *F. graminearum*, the specific mechanism/s of action of these genes during non-self-interactions of the pathogen and the biological role/s associated with species included in the FHBSC. We also shed some light on the KP4L protein family’s diversity in the genus *Fusarium* and identified a new class of KP4L proteins in the species of the *Solani*, *Fujikuroi* and *Oxysporum* species complexes that may have different biological functions.

## Data Availability

Protein accession numbers analyzed during the present study are available in public databases (National Center for Biotechnology Information—NCBI).

## Supplementary Materials

The following supporting information can be downloaded at: https://www.mdpi.com/article/10.3390/jof8090968/s1, Figure S1: Dual cultures of *Fusarium graminearum* with *Trichoderma gamsii* on PDA, Table S1: Primer sequences used for gene expression analysis of *Fusarium graminearum* KP4L-encoding genes (5′ to 3′), Table S2: *Fusarium* spp. and *Trichoderma gamsii* genomic platform, Table S3: Percentages of sequence similarities among *Fusarium* spp. KP4L proteins and with the Ustilago maydis KP4 (UmKP4) protein.

## References

[1] Summerell B.A. (2019). Resolving *Fusarium*: Current Status of the Genus. Annu. Rev. Phytopathol..

[2] Rampersad S. (2020). Pathogenomics and Management of *Fusarium* Diseases in Plants. Pathogens.

[3] Torres A., Palacios S., Yerkovich N., Palazzini J., Battilani P., Leslie J., Logrieco A., Chulze S. (2019). *Fusarium* head blight and mycotoxins in wheat: Prevention and control strategies across the food chain. World Mycotoxin J..

[4] Champeil A., Doré T., Fourbet J. (2004). *Fusarium* head blight: Epidemiological origin of the effects of cultural practices on head blight attacks and the production of mycotoxins by *Fusarium* in wheat grains. Plant Sci..

[5] Morcia C., Tumino G., Gasparo G., Ceresoli C., Fattorini C., Ghizzoni R., Carnevali P., Terzi V. (2020). Moving from qPCR to Chip Digital PCR Assays for Tracking of some *Fusarium* Species Causing *Fusarium* Head Blight in Cereals. Microorganisms.

[6] Sarrocco S., Vannacci G. (2018). Preharvest application of beneficial fungi as a strategy to prevent postharvest mycotoxin contamination: A review. Crop Prot..

[7] Sarrocco S., Valenti F., Manfredini S., Esteban P., Bernardi R., Puntoni G., Baroncelli R., Haidukowski M., Moretti A., Vannacci G. (2019). Is Exploitation Competition Involved in a Multitrophic Strategy for the Biocontrol of Fusarium Head Blight?. Phytopathology.

[8] Matarese F., Sarrocco S., Gruber S., Seidl-Seiboth V., Vannacci G. (2012). Biocontrol of *Fusarium* head blight: Interactions between *Trichoderma* and mycotoxigenic *Fusarium*. Microbiology.

[9] Sarrocco S., Matarese F., Moncini L., Pachetti G., Ritieni A., Moretti A., Vannacci G. (2013). Biocontrol of Fusarium head blight by spike application of Trichoderma gamsii. J. Plant Pathol..

[10] Sarrocco S., Esteban P., Vicente I., Bernardi R., Plainchamp T., Domenichini S., Puntoni G., Baroncelli R., Vannacci G., Dufresne M. (2020). Straw Competition and Wheat Root Endophytism of *Trichoderma gamsii* T6085 as Useful Traits in the Biological Control of Fusarium Head Blight. Phytopathology.

[11] Zapparata A., Baroncelli R., Durling M.B., Kubicek C.P., Karlsson M., Vannacci G., Sarrocco S. (2021). Fungal cross-talk: An integrated approach to study distance communication. Fungal Genet. Biol..

[12] Gu F., Khimani A., Rane S.G., Flurkey W.H., Bozarth R.F., Smith T.J. (1995). Structure and function of a virally encoded fungal toxin from Ustilago maydis: A fungal and mammalian Ca^2+^ channel inhibitor. Structure.

[13] Gage M.J., Bruenn J., Fischer M., Sanders D., Smith T.J. (2001). KP4 fungal toxin inhibits growth in Ustilago maydis by blocking calcium uptake. Mol. Microbiol..

[14] Gage M.J., Rane S.G., Hockerman G.H., Smith T.J. (2002). The Virally Encoded Fungal Toxin KP4 Specifically Blocks L-Type Voltage-Gated Calcium Channels. Mol. Pharmacol..

[15] Puhalla J.E. (1968). Compatibility Reactions on Solid Medium and Interstrain Inhibition in *Ustilago Maydis*. Genetics.

[16] Herring A.J., Bevan E.A. (1974). Virus-like Particles Associated with the Double-stranded RNA Species Found in Killer and Sensitive Strains of the Yeast *Saccharomyces cerevisiae*. J. Gen. Virol..

[17] Stark M., Boyd A. (1986). The killer toxin of Kluyveromyces lactis: Characterization of the toxin subunits and identification of the genes which encode them. EMBO J..

[18] Brown D.W. (2011). The KP4 killer protein gene family. Curr. Genet..

[19] Hayman G.T., Bolen P.L. (1991). Linear DNA plasmids of *Pichia inositovora* are associated with a novel killer toxin activity. Curr. Genet..

[20] Lu S., Faris J. (2018). *Fusarium graminearum* KP4-like proteins possess root growth-inhibiting activity against wheat and potentially contribute to fungal virulence in seedling rot. Fungal Genet. Biol..

[21] Lu S., Edwards M.C. (2016). Genome-Wide Analysis of Small Secreted Cysteine-Rich Proteins Identifies Candidate Effector Proteins Potentially Involved in *Fusarium graminearum*–Wheat Interactions. Phytopathology.

[22] Seidl V., Huemer B., Seiboth B., Kubicek C.P. (2005). A complete survey of *Trichoderma* chitinases reveals three distinct subgroups of family 18 chitinases. FEBS J..

[23] Tzelepis G., Karlsson M. (2019). Killer toxin-like chitinases in filamentous fungi: Structure, regulation and potential roles in fungal biology. Fungal Biol. Rev..

[24] Zapparata A., Da Lio D., Somma S., Muñoz I.V., Malfatti L., Vannacci G., Moretti A., Baroncelli R., Sarrocco S. (2017). Genome Sequence of *Fusarium graminearum* ITEM 124 (ATCC 56091), a Mycotoxigenic Plant Pathogen. Genome Announc..

[25] Vicente I., Baroncelli R., Morán-Diez M.E., Bernardi R., Puntoni G., Hermosa R., Monte E., Vannacci G., Sarrocco S. (2020). Combined Comparative Genomics and Gene Expression Analyses Provide Insights into the Terpene Synthases Inventory in *Trichoderma*. Microorganisms.

[26] Logemann J., Schell J., Willmitzer L. (1987). Improved method for the isolation of RNA from plant tissues. Anal. Biochem..

[27] Bustin S.A., Benes V., Garson J.A., Hellemans J., Huggett J., Kubista M., Mueller R., Nolan T., Pfaffl M.W., Shipley G.L. (2009). The MIQE Guidelines: Minimum Information for Publication of Quantitative Real-Time PCR Experiments. Clin. Chem..

[28] Livak K.J., Schmittgen T.D. (2001). Analysis of relative gene expression data using real-time quantitative PCR and the 2^−ΔΔCT^ Method. Methods.

[29] National Center for Biotechnology Information (NCBI). https://www.ncbi.nlm.nih.gov/.

[30] Baroncelli R., Zapparata A., Piaggeschi G., Sarrocco S., Vannacci G. (2016). Draft Whole-Genome Sequence of *Trichoderma gamsii* T6085, a Promising Biocontrol Agent of *Fusarium* Head Blight on Wheat. Genome Announc..

[31] Lysøe E., Harris L.J., Walkowiak S., Subramaniam R., Divon H.H., Riiser E.S., Llorens C., Gabaldón T., Kistler H.C., Jonkers W. (2014). The Genome of the Generalist Plant Pathogen *Fusarium avenaceum* Is Enriched with Genes Involved in Redox, Signaling and Secondary Metabolism. PLoS ONE.

[32] Yang S., Coleman J.J., Vinatzer B.A. (2022). Genome Resource: Draft Genome of *Fusarium avenaceum*, Strain F156N33, isolated from the Atmosphere Above Virginia and Annotated Based on RNA Sequencing Data. Plant Dis..

[33] Cuomo C.A., Güldener U., Xu J.-R., Trail F., Turgeon B.G., Di Pietro A., Walton J.D., Ma L.-J., Baker S.E., Rep M. (2007). The *Fusarium graminearum* Genome Reveals a Link Between Localized Polymorphism and Pathogen Specialization. Science.

[34] Urban M., King R., Andongabo A., Maheswari U., Pedro H., Kersey P., Hammond-Kosack K. (2016). First Draft Genome Sequence of a UK Strain (UK99) of *Fusarium culmorum*. Genome Announc..

[35] Schmidt R., Durling M.B., de Jager V., Menezes R.C., Nordkvist E., Svatoš A., Dubey M., Lauterbach L., Dickschat J.S., Karlsson M. (2018). Deciphering the genome and secondary metabolome of the plant pathogen *Fusarium culmorum*. FEMS Microbiol. Ecol..

[36] Lysøe E., Frandsen R.J.N., Divon H.H., Terzi V., Orrù L., Lamontanara A., Kolseth A.-K., Nielsen K.F., Thrane U. (2016). Draft genome sequence and chemical profiling of *Fusarium langsethiae*, an emerging producer of type A trichothecenes. Int. J. Food Microbiol..

[37] Proctor R.H., McCormick S.P., Kim H.-S., Cardoza R.E., Stanley A.M., Lindo L., Kelly A., Brown D.W., Lee T., Vaughan M.M. (2018). Evolution of structural diversity of trichothecenes, a family of toxins produced by plant pathogenic and entomopathogenic fungi. PLoS Pathog..

[38] O’Donnell K., Ward J.T., Geiser D.M., Kistler H.C., Aoki T. (2004). Genealogical concordance between the mating type locus and seven other nuclear genes supports formal recognition of nine phylogenetically distinct species within the *Fusarium graminearum* clade. Fungal Genet. Biol..

[39] Gardiner D.M., McDonald M.C., Covarelli L., Solomon P.S., Rusu A.G., Marshall M., Kazan K., Chakraborty S., McDonald B., Manners J.M. (2012). Comparative Pathogenomics Reveals Horizontally Acquired Novel Virulence Genes in Fungi Infecting Cereal Hosts. PLoS Pathog..

[40] Vanheule A., Audenaert K., Warris S., van de Geest H., Schijlen E., Höfte M., De Saeger S., Haesaert G., Waalwijk C., van der Lee T. (2016). Living apart together: Crosstalk between the core and supernumerary genomes in a fungal plant pathogen. BMC Genom..

[41] Ma L.-J., Van Der Does H.C., Borkovich K.A., Coleman J.J., Daboussi M.-J., Di Pietro A., Dufresne M., Freitag M., Grabherr M., Henrissat B. (2010). Comparative genomics reveals mobile pathogenicity chromosomes in *Fusarium*. Nature.

[42] Niehaus E.-M., Münsterkötter M., Proctor R.H., Brown D.W., Sharon A., Idan Y., Oren-Young L., Sieber C.M., Novák O., Pěnčík A. (2016). Comparative “Omics” of the *Fusarium fujikuroi* Species Complex Highlights Differences in Genetic Potential and Metabolite Synthesis. Genome Biol. Evol..

[43] Wiemann P., Sieber C.M., von Bargen K.W., Studt L., Niehaus E.-M., Espino J.J., Huß K., Michielse C.B., Albermann S., Wagner D. (2013). Deciphering the Cryptic Genome: Genome-wide Analyses of the Rice Pathogen *Fusarium fujikuroi* Reveal Complex Regulation of Secondary Metabolism and Novel Metabolites. PLoS Pathog..

[44] Fokkens L., Guo L., Dora S., Wang B., Ye K., Sánchez-Rodríguez C., Croll D. (2020). A Chromosome-Scale Genome Assembly for the *Fusarium oxysporum* Strain Fo5176 To Establish a Model *Arabidopsis*-Fungal Pathosystem. G3 Genes|Genomes|Genetics.

[45] Wang B., Yu H., Jia Y., Dong Q., Steinberg C., Alabouvette C.L., Edel-Hermann V., Kistler H.C., Ye K., Ma L.-J. (2020). Chromosome-Scale Genome Assembly of *Fusarium oxysporum* Strain Fo47, a Fungal Endophyte and Biocontrol Agent. Mol. Plant-Microbe Interact..

[46] Ma L.-J., Shea T., Young S., Zeng Q., Kistler H.C. (2014). Genome Sequence of Fusarium oxysporum f. sp. *melonis* Strain NRRL 26406, a Fungus Causing Wilt Disease on Melon. Genome Announc..

[47] DeIulio G.A., Guo L., Zhang Y., Goldberg J.M., Kistler H.C., Ma L.-J. (2018). Kinome Expansion in the *Fusarium oxysporum* Species Complex Driven by Accessory Chromosomes. mSphere.

[48] Williams A.H., Sharma M., Thatcher L.F., Azam S., Hane J.K., Sperschneider J., Kidd B.N., Anderson J.P., Ghosh R., Garg G. (2016). Comparative genomics and prediction of conditionally dispensable sequences in legume–infecting *Fusarium oxysporum formae speciales* facilitates identification of candidate effectors. BMC Genom..

[49] Coleman J.J., Rounsley S.D., Rodriguez-Carres M., Kuo A., Wasmann C.C., Grimwood J., Schmutz J., Taga M., White G.J., Zhou S. (2009). The Genome of *Nectria haematococca*: Contribution of Supernumerary Chromosomes to Gene Expansion. PLoS Genet..

[50] Emms D.M., Kelly S. (2019). OrthoFinder: Phylogenetic orthology inference for comparative genomics. Genome Biol..

[51] Rozewicki J., Li S., Amada K.M., Standley D.M., Katoh K. (2019). MAFFT-DASH: Integrated protein sequence and structural alignment. Nucleic Acids Res..

[52] Geneious Prime Geneious Prime® v2022.1.1. http://www.geneious.com/.

[53] Price M.N., Dehal P.S., Arkin A.P. (2010). FastTree 2—Approximately Maximum-Likelihood Trees for Large Alignments. PLoS ONE.

[54] Park C.-M., Bruenn J.A., Ganesa C., Flurkey W.F., Bozarth R.F., Koltin Y. (1994). Structure and heterologous expression of the *Ustilago maydis* viral toxin KP4. Mol. Microbiol..

[55] Jones P., Binns D., Chang H.-Y., Fraser M., Li W., McAnulla C., McWilliam H., Maslen J., Mitchell A., Nuka G. (2014). InterProScan 5: Genome-scale protein function classification. Bioinformatics.

[56] Khan A., Mathelier A. (2017). Intervene: A tool for intersection and visualization of multiple gene or genomic region sets. BMC Bioinform..

[57] Abascal F., Zardoya R., Posada D. (2005). ProtTest: Selection of best-fit models of protein evolution. Bioinformatics.

[58] Whelan S., Goldman N. (2001). A General Empirical Model of Protein Evolution Derived from Multiple Protein Families Using a Maximum-Likelihood Approach. Mol. Biol. Evol..

[59] Xu X., Nicholson P. (2009). Community Ecology of Fungal Pathogens Causing Wheat Head Blight. Annu. Rev. Phytopathol..

[60] Boynton P.J. (2019). The ecology of killer yeasts: Interference competition in natural habitats. Yeast.

[61] Seidl-Seiboth V., Ihrmark K., Druzhinina I.S., Karlsson M., Gupta V.K., Schmoll M., Herrera-Estrella A., Upadhyay R.S., Druzhinina I., Tuohy M.G. (2014). Molecular evolution of *Trichoderma chitinases*. Biotechnology and Biology of Trichoderma.

[62] Allen A., Snyder A.K., Preuss M., Nielsen E.E., Shah D.M., Smith T.J. (2008). Plant defensins and virally encoded fungal toxin KP4 inhibit plant root growth. Planta.

[63] Lutz M.P., Feichtinger G., Défago G., Duffy B. (2003). Mycotoxigenic *Fusarium* and Deoxynivalenol Production Repress Chitinase Gene Expression in the Biocontrol Agent *Trichoderma atroviride* P1. Appl. Environ. Microbiol..

[64] Zhang J., Miao Y., Rahimi M.J., Zhu H., Steindorff A.S., Schiessler S., Cai F., Pang G., Chenthamara K., Xu Y. (2019). Guttation capsules containing hydrogen peroxide: An evolutionarily conserved NADPH oxidase gains a role in wars between related fungi. Environ. Microbiol..

[65] Tzelepis G.D., Melin P., Stenlid J., Jensen D.F., Karlsson M. (2014). Functional analysis of the C-II subgroup killer toxin-like chitinases in the filamentous ascomycete *Aspergillus nidulans*. Fungal Genet. Biol..

