# Supplementary material for: Insights on KP4 Killer Toxin-like Proteins of Fusarium Species in Interspecific Interactions
Source: J Fungi (Basel). 2022 Sep 16;8(9):968. doi: 10.3390/jof8090968 (PMC9506348; doi:10.3390/jof8090968)

Figure S1. Dual cultures of *Fusarium graminearum* and *Trichoderma gamsii* on PDA. Early-sensing phase, 15 mm of distance between colonies; Sensing phase, 5-8 mm of distance between colonies; After-contact phase, 24 h overgrowth. For each early-sensing and sensing sampling, 5 mm of the edge of *F. graminearum* colony were collected from both self- (*F. graminearum* vs itself - control) and non-self-interaction (*F. graminearum* vs *T. gamsii*) plates. For each after-contact sampling, 10 mm of the contact zone between colonies were collected from both self- (*F. graminearum* vs itself - control) and non-self-interaction (*F. graminearum* vs *T. gamsii*) plates. In the non-self-interaction plates, *T. gamsii* T6085 is shown on the left and *F. graminearum* ITEM 124 on the right.

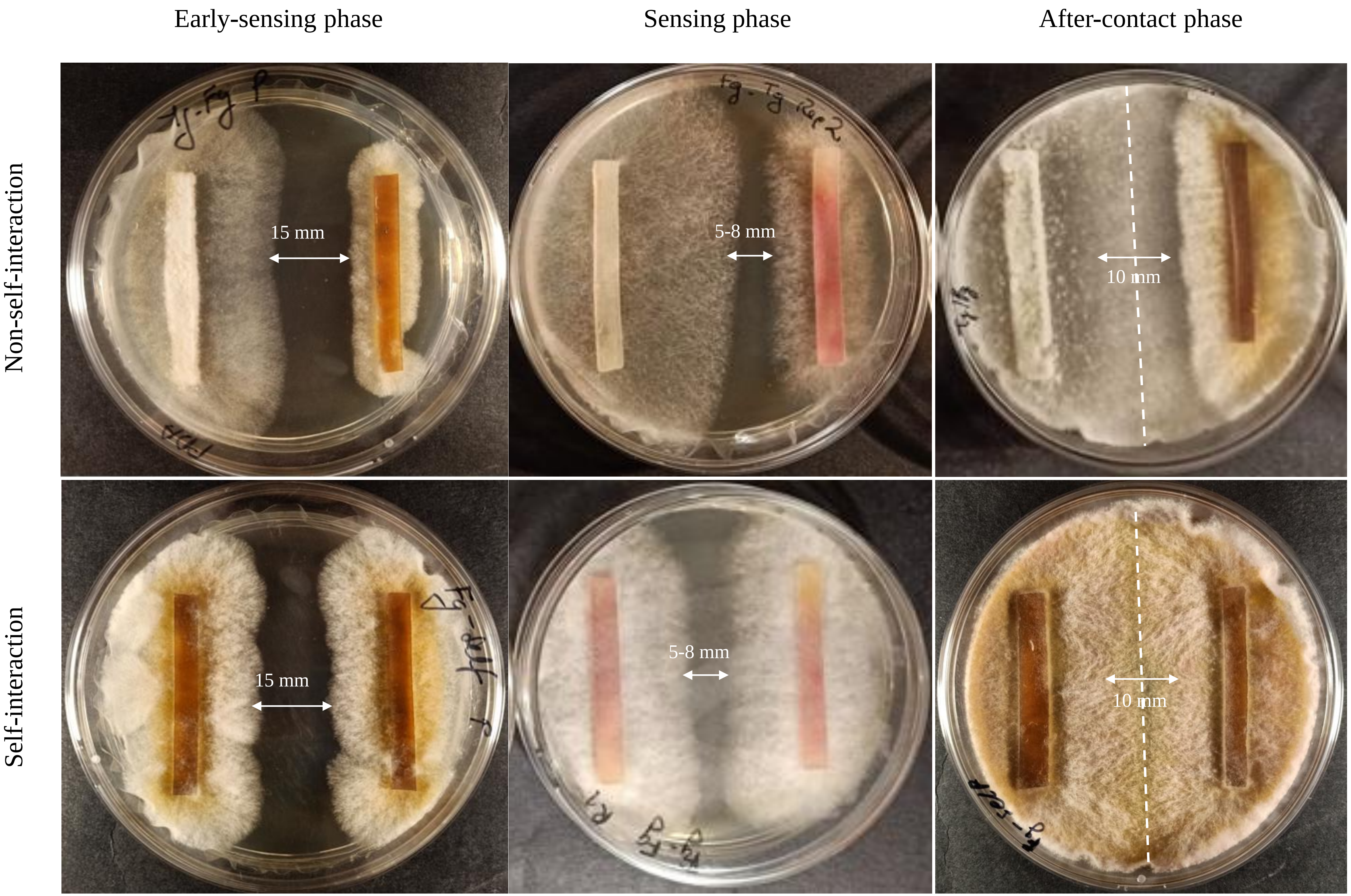

Supplement: Supplementary file 1 [file jof-08-00968-s001.zip › Figure S1.pdf]
