# Supplementary material for: Insights on KP4 Killer Toxin-like Proteins of Fusarium Species in Interspecific Interactions
Source: J Fungi (Basel). 2022 Sep 16;8(9):968. doi: 10.3390/jof8090968 (PMC9506348; doi:10.3390/jof8090968)
Supplement: Supplementary file 1 [file jof-08-00968-s001.zip › Table S1.pdf]

Table S1. Primer sequences used for gene expression analysis of *Fusarium graminearum* KP4L-encoding genes (5’ to 3’).
Primers selected for gene expression are shown in black color.

Name	Sequence	Gene	Amplicon size
ACT-F<br>ACT-R	TCAACGCCCCCGCTTTCTA<br>GATTTCTCGCTCGGCAGTGG	*act*	245 bp
TEF-F<br>TEF-R	CCACGTCGACTCTGGCAAGT<br>CGGCTTTGAGCTTGTCAAGAAC	*tef-1*	155 bp
TUB-F<br>TUB-R	CAAGGTCTCCGACACCGTTGT<br>GACATGACGGCAGAGACAAGGT	*tub*	183 bp
KP4L-1-F<br>KP4L-1-R	TCCTCACTCTCACCACCCTTCT<br>CGGGCTTGTAGTTGTTTCGC	*kp4l-1*	130 bp
KP4L-2-F<br>KP4L-2-R	CGCTCTCGGAATCAACTGTCG<br>CTCATAGTGGCGGTCTCGTGG	*kp4l-2*	112 bp
KP4L-3-F<br>KP4L-3-R	ACCTTCGGCTCTAGCATTGGAG<br>AGCACAGATACTGCCCTGGTTG	*kp4l-3*	131 bp
KP4L-4-F<br>KP4L-4-R	CACCCTTTTGACCCTTGCC<br>TGCTGAACCTCTTGTTGGGATCT	*kp4l-4*	149 bp
