# Supplementary material for: Insights on KP4 Killer Toxin-like Proteins of Fusarium Species in Interspecific Interactions
Source: J Fungi (Basel). 2022 Sep 16;8(9):968. doi: 10.3390/jof8090968 (PMC9506348; doi:10.3390/jof8090968)
Supplement: Supplementary file 1 [file jof-08-00968-s001.zip › Table S2.pdf]

Table S2. *Fusarium* spp. and *Trichoderma gamsii* genomic platform. Complete genome sequences of *Fusarium* spp. and *T. gamsii* were obtained from public databases of NCBI. Whole Genome Sequencing (WGS) Project accessions of each species are listed in the second column. Phylogeny according to O'Donnell et al. [38] and Summerell [1], and references of each strain are shown.

Strain	Phylogeny	WGS Project	Reference
T. gamsiii T6085		JPDN00000000	[30]
F. avenaceum Fa05001	Species complex: Tricincum	JPYM00000000	[31]
F. avenaceum F156N33	Species complex: Tricincum	JAGPUO000000000	[32]
F. graminearum PH-1	Species complex: Sambucinum	AACM00000000	[33]
F. graminearum ITEM 124	Species complex: Sambucinum	NQOC00000000	[24]
F. culmorum Class 2-1B	Species complex: Sambucinum	PVEM00000000	[34]
F. culmorum PV	Species complex: Sambucinum	PVEM00000000	[35]
F. langsethiae Fl201059	Species complex: Sambucinum	JXCE00000000	[36]
F. sporotrichioides NRRL3299	Species complex: Sambucinum	PXOF00000000	[37]
F. austroamericanum NRRL2903	Species complex: Sambucinum	JAAMOD000000000	[38]
F. longipes NRRL20695	Species complex: Sambucinum	PXOG00000000	[37]
F. pseudograminearum CS3096	Species complex: Sambucinum	AFNW00000000	[39]
F. poae 2516	Species complex: Sambucinum	LYXU00000000	[40]
F. verticillioides 7600	Species complex: Fujikuroi Clade: African	AAIM00000000	[41]
F. mangiferae MRC7560	Species complex: Fujikuroi Clade: Asian	FCQH00000000	[42]
F. fujikuroi IMI 58289	Species complex: Fujikuroi Clade: Asian	AGND01000000	[43]
F. proliferatum ET1	Species complex: Fujikuroi Clade: Asian	FJOF00000000	[42]
F. oxysporum Fo5176	Species complex: Oxysporum	JACDXP000000000	[44]
F. oxysporum Fo47	Species complex: Oxysporum	WXUE00000000	[45]
F. oxysporum f. sp. *melonis* NRRL26406	Species complex: Oxysporum	AGNE00000000	[46]
F. oxysporum f. sp. *conglutinans* 54008	Species complex: Oxysporum	AGNF00000000	[47]
F. oxysporum f. sp. *lycopersici* MN25	Species complex: Oxysporum	AGBH00000000	[47]
F. oxysporum f. sp. *vasinfectum* 25433	Species complex: Oxysporum	AGNC00000000	[47]
F. oxysporum NRRL32931	Species complex: Oxysporum	AFML00000000	[47]
F. oxysporum f. sp. *pisi* HDV247	Species complex: Oxysporum	AGBI00000000	[48]
F. oxysporum f. sp. *cubense* NRRL54006	Species complex: Oxysporum	AGND00000000	[47]
F. oxysporum f. sp. *radicis-lycopersici* 26381	Species complex: Oxysporum	AGNB00000000	[47]
F. oxysporum f. sp. *raphani* 54005	Species complex: Oxysporum	AGNG00000000	[47]
F. oxysporum f. sp. *lycopersici* 4287	Species complex: Oxysporum	AAXH00000000	[41]
F. vanettenii 77-13-4	Species complex: Solani Clade: 3	ACJF00000000	[49]
