# Supplementary material for: Insights on KP4 Killer Toxin-like Proteins of Fusarium Species in Interspecific Interactions
Source: J Fungi (Basel). 2022 Sep 16;8(9):968. doi: 10.3390/jof8090968 (PMC9506348; doi:10.3390/jof8090968)
Supplement: Supplementary file 1 [file jof-08-00968-s001.zip › Table S3.pdf]

Table S3. Percentages of sequence similarities among *Fusarium* spp. KP4L proteins and with the *Ustilago maydis* KP4 (UmKP4) protein. (A) Intraspecific variability was calculated by pairwise alignment between proteins from different isolates of the same species. **(B)** Interspecific variability was calculated among proteins from different species considering proteins of either all *Fusarium* spp., only species belonging to the FHBSC, or only species outside the FHBSC. **(C)** Intergeneric variability was calculated by pairwise alignments between the UmKP4 protein and the consensus sequence derived from the alignment of KP4L proteins from either all *Fusarium* spp., only species belonging to the FHBSC, or only species outside the FHBSC.

A

**Intraspecific**	KP4L-0	KP4L-1	KP4L-2	KP4L-3	KP4L-4	KP4L-4 D1	KP4L-4 D2	TEF-1
F. graminearum	.	100	100	100	95	95	95	100
F. avenaceum	.	98	96	97	98	98	99	99.8
F. culmorum	.	100	100	100	100	100	100	99.9
*F. oxysporum**	.	.	.	.	99	99	100	99.8

B

**Interspecific**	KP4L-0	KP4L-1	KP4L-2	KP4L-3	KP4L-4	KP4L-4 D1	KP4L-4 D2	TEF-1
Fusarium spp.	56	94	93	92	70	66	78	92.8
FHBSC	.	99	97	98	83	79	91	95.4
Outside FHBSC	56	.	.	.	70	66	73	96.7

C

**Intergeneric**	KP4L-0	KP4L-1	KP4L-2	KP4L-3	KP4L-4	KP4L-4 D1	KP4L-4 D2
Fusarium spp.	33	39	36	41	.	36	37
FHBSC	.	39	36	41	.	36	37
Outside FHBSC	33	37	35	39	.	30	32

* Fo47, NRRL32931, *F. oxysporum* f. sp. *melonis*, *F. oxysporum* f. sp. *lycopersici*
